# Supplementary material for: Grape Small Auxin Upregulated RNA (SAUR) 041 Is a Candidate Regulator of Berry Size in Grape
Source: Int J Mol Sci. 2021 Oct 30;22(21):11818. doi: 10.3390/ijms222111818 (PMC8584205; doi:10.3390/ijms222111818)
Supplement: Supplementary file 1 [file ijms-22-11818-s001.zip › ijms-1401779-supplementary.pdf]

International Journal of
Molecular Sciences

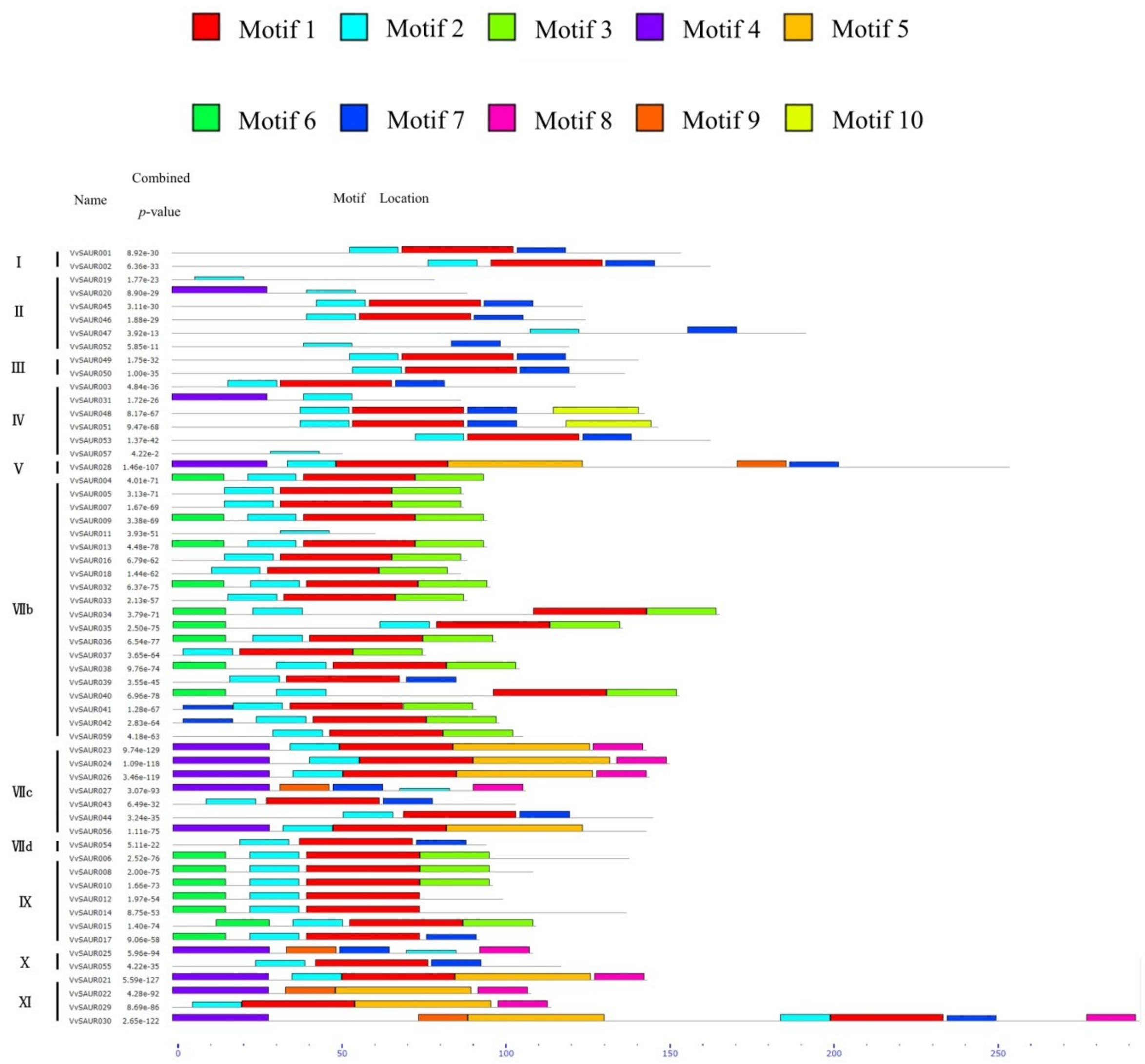

Figure S1. The conservative motif analysis of the *VvSAUR* family. The distribution of motifs in the *VvSAUR* family is represented by rectangles of different colors.

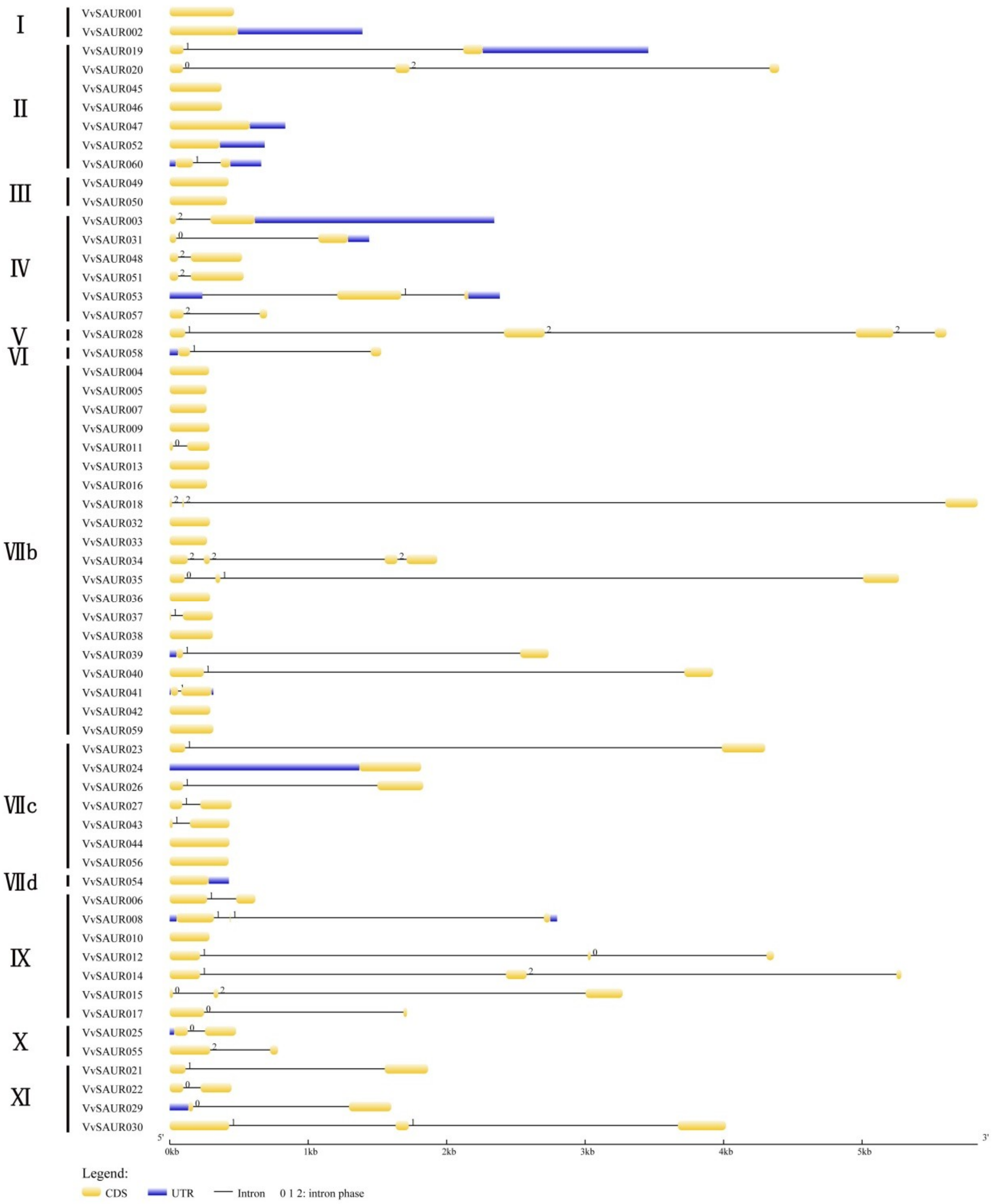

Figure S2. The structural features of the *VvSAUR* family. The blue rectangles represent untranslated regions (UTRs). The yellow round-cornered rectangles represent exons. The black lines represent introns. The number above the lines represents the intron phase.

Table S1. Details of the *VvSAUR* family. The gene locus is available in the National Center for Biotechnology Information Database. Mw, molecular weight; PI, isoelectric point.

Gene locus	Gene symbol	ORF length(bp)	No. of aa	Mw	PI	Chr.	Location	Orientation	Group
GSVIVG01000929001	VvSAUR001	465	155	17397.04	9.62	1	22022632-22023096	forward	Ⅰ
GSVIVG01001263001	VvSAUR002	492	164	19012.29	10.62	2	4821803-4823194	reverse	Ⅰ
GSVIVG01024152001	VvSAUR003	369	123	13585.51	5.61	3	736293-738636	forward	Ⅳ
GSVIVG01024138001	VvSAUR004	285	95	10554.18	6.56	3	858886-859170	reverse	Ⅶb
GSVIVG01024136001	VvSAUR005	267	89	9522.85	5.71	3	864608-864874	forward	Ⅶb
GSVIVG01024135001	VvSAUR006	411	137	15518.07	8.88	3	866406-867024	forward	Ⅸ
GSVIVG01024134001	VvSAUR007	267	89	9536.88	5.71	3	867890-868156	forward	Ⅶb
GSVIVG01024133001	VvSAUR008	324	108	12260.50	8.91	3	869578-872375	forward	Ⅸ
GSVIVG01024131001	VvSAUR009	288	96	10559.18	6.82	3	883689-883976	reverse	Ⅶb
GSVIVG01024130001	VvSAUR010	288	96	10767.51	6.95	3	885397-885684	reverse	Ⅸ
GSVIVG01024129001	VvSAUR011	186	62	6807.89	4.70	3	888666-888953	forward	Ⅶb
GSVIVG01024128001	VvSAUR012	297	99	11306.30	9.63	3	890779-895141	reverse	Ⅸ
GSVIVG01024127001	VvSAUR013	288	96	10496.09	6.55	3	897891-898178	forward	Ⅶb
GSVIVG01024126001	VvSAUR014	408	136	15613.93	9.22	3	898286-903569	reverse	Ⅸ
GSVIVG01024125001	VvSAUR015	327	109	11939.88	6.89	3	905892-909162	forward	Ⅸ
GSVIVG01024123001	VvSAUR016	270	90	9743.06	4.97	3	915230-915499	reverse	Ⅶb
GSVIVG01024122001	VvSAUR017	276	92	10479.22	9.51	3	915998-917711	reverse	Ⅸ
GSVIVG01024120001	VvSAUR018	264	88	9643.04	5.60	3	922119-927953	forward	Ⅶb
GSVIVG01023917001	VvSAUR019	240	80	9292.95	11.33	3	2502521-2505976	forward	Ⅱ
GSVIVG01023916001	VvSAUR020	270	90	10507.66	12.02	3	2508656-2513056	forward	Ⅱ
GSVIVG01023915001	VvSAUR021	429	143	16365.33	9.27	3	2513557-2515422	forward	Ⅺ
GSVIVG01023913001	VvSAUR022	324	108	12236.35	8.68	3	2518586-2519032	forward	Ⅺ
GSVIVG01023912001	VvSAUR023	426	142	16204.15	9.65	3	2520298-2524597	forward	Ⅶc
GSVIVG01023911001	VvSAUR024	447	149	17029.75	9.03	3	2525431-2527246	reverse	Ⅶc
GSVIVG01023910001	VvSAUR025	324	108	12071.35	9.62	3	2528389-2528867	forward	Ⅹ
GSVIVG01023909001	VvSAUR026	429	143	16236.16	9.35	3	2530531-2532361	forward	Ⅶc
GSVIVG01023908001	VvSAUR027	318	106	12068.19	9.51	3	2533266-2533712	reverse	Ⅶc

GSVIVG01023907001	VvSAUR028	765	255	28965.02	9.41	3	2536206-2541814	forward	V
GSVIVG01023881001	VvSAUR029	342	114	12891.88	5.48	3	2749512-2751111	reverse	XI
GSVIVG01023880001	VvSAUR030	873	291	32880.35	5.63	3	2751841-2755856	reverse	XI
GSVIVG01023879001	VvSAUR031	264	88	9888.54	10.23	3	2757523-2758963	forward	IV
GSVIVG01019106001	VvSAUR032	291	97	10860.46	7.82	4	16733927-16734217	reverse	VIIb
GSVIVG01019105001	VvSAUR033	270	90	9990.55	8.64	4	16735502-16735771	reverse	VIIb
GSVIVG01019103001	VvSAUR034	492	164	18532.53	8.54	4	16738840-16740771	reverse	VIIb
GSVIVG01019101001	VvSAUR035	405	135	15287.92	9.67	4	16744811-16750076	reverse	VIIb
GSVIVG01019099001	VvSAUR036	291	97	10809.53	7.84	4	16755874-16756164	reverse	VIIb
GSVIVG01019097001	VvSAUR037	228	76	8361.52	6.26	4	16763888-16764199	reverse	VIIb
GSVIVG01019096001	VvSAUR038	312	104	11472.27	9.25	4	16765755-16766066	reverse	VIIb
GSVIVG01019095001	VvSAUR039	255	85	9441.86	7.95	4	16767618-16770353	reverse	VIIb
GSVIVG01019094001	VvSAUR040	456	152	16871.48	8.89	4	16772358-16776281	reverse	VIIb
GSVIVG01019093001	VvSAUR041	273	91	10019.53	6.25	4	16779757-16780072	reverse	VIIb
GSVIVG01019092001	VvSAUR042	294	98	10962.60	6.16	4	16782060-16782353	reverse	VIIb
GSVIVG01031508001	VvSAUR043	309	103	11635.28	5.76	6	17809104-17809535	forward	VIIc
GSVIVG01031507001	VvSAUR044	432	144	16135.55	6.83	6	17828336-17828767	forward	VIIc
GSVIVG01025708001	VvSAUR045	375	125	14244.64	8.84	8	12797230-12797604	forward	II
GSVIVG01033868001	VvSAUR046	378	126	13881.91	6.29	8	17142119-17142496	reverse	II
GSVIVG01016698001	VvSAUR047	579	193	21144.01	6.21	9	445796-446630	forward	II
GSVIVG01029567001	VvSAUR048	432	144	16284.00	6.10	9	21457323-21457844	forward	IV
GSVIVG01029566001	VvSAUR049	426	142	16737.43	9.37	9	21482997-21483422	reverse	III
GSVIVG01029559001	VvSAUR050	414	138	16094.85	9.30	9	21569331-21569744	forward	III
GSVIVG01029558001	VvSAUR051	444	148	16771.60	6.43	9	21572109-21572642	reverse	IV
GSVIVG01015021001	VvSAUR052	363	121	13265.03	5.60	11	516935-517620	forward	II
GSVIVG01015023001	VvSAUR053	492	164	18227.53	5.22	11	528067-530450	reverse	IV
GSVIVG01020838001	VvSAUR054	282	94	10594.48	9.23	12	1253357-1253784	reverse	VIId
GSVIVG01038622001	VvSAUR055	351	117	13282.46	6.06	16	21394851-21395632	reverse	X
GSVIVG01008695001	VvSAUR056	426	142	15802.62	9.83	18	1262260-1262685	forward	VIIc

GSVIVG01009870001	VvSAUR057	156	52	6102.37	11.79	18	11959701-11960404	forward	Ⅳ
GSVIVG01009872001	VvSAUR058	165	55	5987.21	10.31	18	11986451-11987978	reverse	Ⅵ
GSVIVG01009875001	VvSAUR059	315	105	11720.72	8.85	18	12002966-12003280	forward	Ⅷb
GSVIVG01036807001	VvSAUR060	198	66	7502.57	10.03	19	22279115-22279775	forward	Ⅱ

Table S2. Normal expression sequences of 10 motifs identified. Sites represent the time of motif appeared.

Motif	Sequences	Sites	Length(aa)
Motif1	[KR][KR]RF[VM][VI]P[IL][SVE]YL[NKS][HN][PN][SI]F[QR][NEKA]L[LF][SER][QME][AS]EEE[FY]GF[DNQ][HQS][PE][GM][GP]	48	34
Motif2	[PTSA][ASKT][NDVE][VA][PD]KG[HY][FLV][AVP]VYVG[ED]	57	15
Motif3	GLTIPC[KTREN]E[ED][AT]F[IL][DN][LV][TA][SC][RSQ]L[NQS][ASC]S	22	21
Motif4	MI[SN][PA]KKLI[KR]MAR[KEN]WQ[KR][MY]AA[ILM][RGK]RKRI[SI][LS][PQ]R	12	29
Motif5	I[TI]LP[CYF]D[SAL][VFA][FT][ML][EDQ]Y[IAVM][IVL][PSF][LFM][IV][QG][RQ][GH][VM][AS][KE][DE][LIVM]E[KR]AL[LI][MFVTI]S[ILV][AVG][TYS][STD][RNTQC][CS][SL][SLQC][SLF]	9	41
Motif6	M[GAS][IFN][RHA][LF][PQD][SRGL][IMV][IVLG][PQN]AK[QK][IVF][LVPF][RKQ]	16	16
Motif7	[TKVQ][ILV]P[CS][EDRV][VEISP][EVST][EFV][FMLIA][EKRQ][YKER][IVL][LY][KSG][LMEV]	24	15
Motif8	[SFI][HQC]Q[EG][QH][MTSNI][NGS][ESQH][QRP]LL[LVF]C[ASGR][YFC]	9	15
Motif9	F[KQE]MSEEE[FY]G[LI][PQ][SR][DTN]GP	5	15
Motif10	NAEKEMIGCCSSSDCELT-PSHHPQMC	2	26

Table S3. The orthologous *SAUR* genes in grape, *Arabidopsis* and tomato genome.

SAUR Name1	SAUR Name2
VvSAUR002	AtSAUR045
VvSAUR003	AtSAUR004
VvSAUR003	AtSAUR018
VvSAUR003	AtSAUR067
VvSAUR021	AtSAUR006
VvSAUR021	AtSAUR007
VvSAUR021	AtSAUR008
VvSAUR021	AtSAUR009
VvSAUR021	AtSAUR012
VvSAUR021	AtSAUR013
VvSAUR021	AtSAUR076
VvSAUR023	AtSAUR006
VvSAUR023	AtSAUR007
VvSAUR023	AtSAUR008
VvSAUR023	AtSAUR009
VvSAUR023	AtSAUR012
VvSAUR023	AtSAUR013
VvSAUR023	AtSAUR076
VvSAUR032	AtSAUR059
VvSAUR033	AtSAUR059
VvSAUR036	AtSAUR059
VvSAUR037	AtSAUR068
VvSAUR037	AtSAUR069
VvSAUR037	AtSAUR070
VvSAUR037	AtSAUR071
VvSAUR037	AtSAUR072
VvSAUR037	AtSAUR073
VvSAUR039	AtSAUR023
VvSAUR041	AtSAUR058
VvSAUR043	AtSAUR027
VvSAUR044	AtSAUR027
VvSAUR045	AtSAUR030
VvSAUR046	AtSAUR029
VvSAUR048	AtSAUR026
VvSAUR049	AtSAUR041
VvSAUR050	AtSAUR041
VvSAUR051	AtSAUR026
VvSAUR052	AtSAUR002
VvSAUR052	AtSAUR075
VvSAUR053	AtSAUR042
VvSAUR055	AtSAUR032
VvSAUR001	SlSAUR062
VvSAUR002	SlSAUR071
VvSAUR003	SlSAUR003
VvSAUR018	SlSAUR101
VvSAUR021	SlSAUR047
VvSAUR024	SlSAUR044
VvSAUR036	SlSAUR084
VvSAUR041	SlSAUR028
VvSAUR043	SlSAUR063
VvSAUR044	SlSAUR063
VvSAUR045	SlSAUR072
VvSAUR046	SlSAUR086
VvSAUR048	SlSAUR041
VvSAUR051	SlSAUR041

VvSAUR052	SlSAUR066
VvSAUR053	SlSAUR065
VvSAUR054	SlSAUR067
VvSAUR055	SlSAUR036

Table S4. The paralogous *SAUR* genes in grape and *Arabidopsis* genome.

SAUR Name1	SAUR Name2
AtSAUR002	AtSAUR075
AtSAUR004	AtSAUR018
AtSAUR004	AtSAUR067
AtSAUR006	AtSAUR007
AtSAUR006	AtSAUR008
AtSAUR006	AtSAUR009
AtSAUR006	AtSAUR012
AtSAUR006	AtSAUR013
AtSAUR006	AtSAUR076
AtSAUR007	AtSAUR008
AtSAUR007	AtSAUR009
AtSAUR007	AtSAUR012
AtSAUR007	AtSAUR013
AtSAUR007	AtSAUR076
AtSAUR008	AtSAUR009
AtSAUR008	AtSAUR012
AtSAUR008	AtSAUR013
AtSAUR008	AtSAUR076
AtSAUR009	AtSAUR012
AtSAUR009	AtSAUR013
AtSAUR009	AtSAUR076
AtSAUR012	AtSAUR013
AtSAUR012	AtSAUR076
AtSAUR013	AtSAUR076
AtSAUR018	AtSAUR067
AtSAUR068	AtSAUR069
AtSAUR068	AtSAUR070
AtSAUR068	AtSAUR071
AtSAUR068	AtSAUR072
AtSAUR068	AtSAUR073
AtSAUR069	AtSAUR070
AtSAUR069	AtSAUR071
AtSAUR069	AtSAUR072
AtSAUR069	AtSAUR073
AtSAUR070	AtSAUR071
AtSAUR070	AtSAUR072
AtSAUR070	AtSAUR073
AtSAUR071	AtSAUR072
AtSAUR071	AtSAUR073
AtSAUR072	AtSAUR073
VvSAUR021	VvSAUR023
VvSAUR032	VvSAUR033
VvSAUR032	VvSAUR036
VvSAUR033	VvSAUR036
VvSAUR043	VvSAUR044
VvSAUR048	VvSAUR051
VvSAUR049	VvSAUR050

Table S5. List of primers used for qRT-PCR and construction of recombinant plasmid.

Gene name	Gene identifier	Forward primer (5'-3')	Reverse primer (5'-3')
VvSAUR041-pB221-GFP	GSVIVG01019093001	TCTAGAATGGG-TATTCGTTTGCCCTCA	GTCGACTTATGAGGCAC-TCAATTGAGA
VvSAUR041-3301	GSVIVG01019093001	CCATGGATGGG-TATTCGTTTGCCCTCA	AGATCTTTATGAGGCAC-TCAATTGAGA
VvSAUR041-pART-CAM-EGFP	GSVIVG01019093001	CTCGAGATGGG-TATTCGTTTGCCCTCA	GAATTCTTATGAGGCAC-TCAATTGAGA
VvSAUR041-qRT	GSVIVG01019093001	GCAG-TCTGTCCATATTAGAAG	GTTAGACCACCCA-TAGAATGA
SlActin	Q96483	TGTCCCTATCTAC-GAGGGTTATGC	AGTTAAATCACGACCAG-CAAGAT
AtUBQ3	AT5G03240	CGGAAAGAC-CATTACTCTGGA	CAAGTGTGCGAC-CATCCTCAA
